# Exploring the Influence of Oral Health on Pregnancy Outcomes: A Narrative Review: Oral Health and Pregnancy Outcomes

**DOI:** 10.1155/ghe3/9304496

**Published:** 2025-07-18

**Authors:** Muhammad Mohsin Javaid, Samina Naeem Khalid, Shahzad Ali Khan, Hina Nasim, Mudassar Mushtaq Jawad Abbasi, Shahzad Ahmad, Shazia Iqbal, Muhammad Farooq Umer

**Affiliations:** ^1^Department of Public Health, Health Services Academy, Islamabad, Pakistan; ^2^Department of Maternal, Neonatal & Child Health, Health Services Academy, Islamabad, Pakistan; ^3^Department of Oral Biology, School of Dentistry/SZABMU, Islamabad, Pakistan; ^4^Faculty of Medicine and Health Sciences, The University of Buckingham, Buckingham, UK; ^5^Department of Preventive Dental Sciences, College of Dentistry, King Faisal University, Hofuf 31982, AlAhsa, Saudi Arabia

**Keywords:** adverse pregnancy outcomes, low birth weight, oral health, periodontitis, pregnancy, preterm birth

## Abstract

Oral health significantly impacts overall health, with increasing evidence linking poor oral health to adverse pregnancy outcomes. Periodontal disease, a chronic gum condition, is associated with preterm birth, low birth weight, and preeclampsia. Systemic inflammation and bacterial translocation are proposed mechanisms connecting periodontal disease to pregnancy complications. Maternal immune responses may be impaired, increasing systemic inflammation, and triggering preterm labor. In addition, oral bacteria may reach the uterus, causing localized inflammation and poor outcomes. Intervention trials show mixed results—some small studies report improved birth outcomes after periodontal treatment, while larger trials show no significant effect. These discrepancies highlight the need for further research on patient subgroups, disease categories, and treatment strategies. Prenatal care should emphasize preventive dental care, including regular checkups, good oral hygiene, and treatment of periodontal disease. Addressing oral health during pregnancy can improve outcomes for both mother and child. Future studies should explore the oral-systemic connection, host susceptibility, and effective interventions for at-risk individuals. Comprehensive oral health care during pregnancy offers the potential to reduce adverse outcomes and promote better maternal and fetal health.

## 1. Introduction

Oral health is an essential yet often overlooked component of prenatal care. Pregnancy brings about hormonal changes that can significantly impact the oral cavity, increasing susceptibility to conditions such as gingivitis, periodontitis, dental caries, and other oral health issues [1]. Periodontitis is an infectious inflammatory disease that affects the periodontal tissues (gingivae, alveolar bone, periodontal ligament, and cementum). The process begins with biofilm buildup on the tooth's sensitive surfaces. Later on, simple inflammation of the gingiva (gingivitis) can cause periodontitis due to a shift in the microbiota. Gingivitis, characterized by inflamed and bleeding gums, affects a substantial number of pregnant women, with studies from India reporting prevalence rates as high as 71.9% [2]. Meanwhile, periodontitis, a more severe form of gum disease, is also prevalent during pregnancy, with studies in Indonesia indicating that 34% of the pregnant women exhibit periodontal pockets measuring 3–5 mm in depth [3]. Dental caries, another common concern, affects a significant proportion of pregnant women globally, with prevalence rates reported as 41.5% in studies conducted in Brazil [4]. These conditions not only exacerbate pre-existing oral health issues but also pose risks to both maternal and fetal health [5].

Despite common misunderstandings, it is safe to get restorative and preventative dental care while pregnant. If necessary, diagnostic radiography might be done after first trimester. Both anesthetics (like lidocaine) and analgesics (like paracetamol) are regarded as safe. Antibacterial medications such as ampicillin, amoxicillin, some cephalosporines, and macrolides may be prescribed in case of an infection. During the first trimester, when the fetus is most vulnerable to severe abnormalities (teratogenesis), organogenesis occurs. The second trimester, which runs from weeks 17 to 28, is the best time to have dental work done. On the other hand, severe pain or infections necessitate immediate dental intervention, and emergency care can be provided throughout the whole pregnancy. Healthcare professionals' awareness and adherence to prenatal oral health recommendations are critical to providing complete treatment for pregnant women, considerably improving their overall health and pregnancy outcomes [6, 7].

Poor oral health during pregnancy has major consequences, including preterm birth, low birth weight, and preeclampsia [8]. For instance, periodontitis can raise the risk of preterm delivery by causing an inflammatory reaction that releases prostaglandins and cytokines, which are linked to labor induction [9]. Research suggests that up to 68.7% of women with periodontitis had low birth weight babies and 62.5% had preterm babies. The transfer of harmful microorganisms to the fetus–placenta unit and the subsequent immune response are considered crucial factors causing these negative results [10]. Moreover, untreated dental infections, such as untreated dental caries or abscesses, can potentially lead to systemic infections that may adversely affect both maternal and fetal health [11]. In addition, gingival hyperplasia, a condition characterized by overgrowth of gum tissue, can occur due to hormonal changes during pregnancy, leading to discomfort and difficulty in maintaining oral hygiene [12]. Another study conducted in Italy found that poor maternal dental hygiene, periodontal disease, and excessive sugar consumption greatly increase the risk of preterm delivery, low birth weight, gestational diabetes, and preeclampsia. The findings revealed that a lack of fluoride knowledge and insufficient dental care contribute to systemic inflammation and infections, which negatively impact pregnancy outcomes [13].

Pregnancy outcomes can be considerably enhanced by including dental healthcare in standard prenatal care. Effective treatment of periodontal disease during pregnancy has been shown in studies to lower the risk of preterm deliveries and low birth weight babies [14]. Dental procedures, such as thorough cleanings and the resolution of any current oral health problems, are advantageous and safe to have during pregnancy [15]. It is essential to teach expectant mothers the value of maintaining excellent oral hygiene, which includes regular dental checkups, brushing, and flossing. To offer pregnant patients comprehensive treatment that meets their dental and general health needs, healthcare professionals should be well-versed in prenatal oral health recommendations, especially those in obstetrics.

The objectives of this narrative review are to describe common oral conditions associated with pregnancy and to provide an overview summary of the available evidence relevant to adverse pregnancy outcomes associated with poor oral health. Moreover, this manuscript also highlights pertinent literature exploring the influence of oral health on pregnancy outcomes.

## 2. Oral Conditions Commonly Associated With Pregnancy

**Figure 1:**
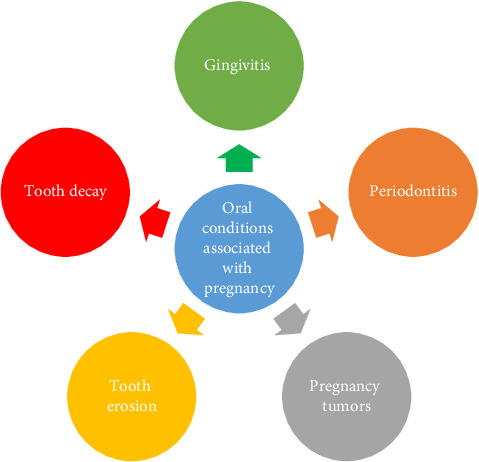
Common oral conditions associated with pregnancy.

Due to hormonal changes, women may suffer from a variety of oral health issues during pregnancy. Gingivitis during pregnancy is a frequent condition marked by swollen, bleeding, and irritated gums. It can worsen into periodontitis, a more serious gum disease that can result in tooth loss and unfavorable pregnancy outcomes if treatment is not received. Pregnancy tumors, sometimes called pyogenic granulomas, are benign growths on certain women's gums that usually go away after giving birth. Too much acid in the mouth from morning sickness and sugar-filled meal cravings might make tooth decay more likely. Hormonal shifts can also result in dry mouth, which lowers salivary flow and raises the risk of cavities and oral infections [16] (Figure 1).

### 2.1. Pregnancy-Induced Gingivitis

Pregnancy is a transforming phase marked by several physiological changes, including those affecting dental health. Gingivitis is a frequent pregnancy ailment that causes inflammation of the gums. Studies indicate that hormonal variations, notably elevated levels of progesterone, can aggravate the body's response to dental plaque, leading to an increased risk of gingivitis in pregnant women [17]. A study conducted in Nigeria reported that prevalence of the gingivitis in pregnant women was 85.2% [18]. Likewise, a study conducted in Senegal reported a gingivitis prevalence of 88.2% among pregnant women [19]. A study conducted in Pakistan also reported a higher prevalence (30.6%) of gingivitis among pregnant women as well [20]. Expectant mothers must practice proper oral hygiene and seek frequent dental care to minimize and lessen the consequences of gingivitis during this key period.

### 2.2. Pregnancy-Induced Periodontitis

Pregnancy poses a significant risk for periodontitis, a severe type of gum disease marked by inflammation and infection of the tooth's supporting tissues. Both the growing fetus and the expecting mother are at serious health danger from this illness. Studies show that the body's reaction to oral bacteria can be influenced by the hormonal changes during pregnancy, especially the elevated levels of progesterone and estrogen. Pregnant women may be more susceptible to developing periodontitis as a result of this hormonal change [10, 21, 22].

Several studies from different parts of the world have highlighted the prevalence of periodontitis in pregnant women. For example, a comprehensive analysis carried out in China revealed that up to 40% of pregnant women had periodontitis. This study highlights the substantial number of pregnant women in China who suffer from this illness, emphasizing the need for improved oral health education and preventative strategies for this population [23]. Comparably, a research carried out in Rwanda discovered that 60.5% of pregnant women had periodontitis, an even greater incidence of the condition. Given the high incidence rate, periodontitis appears to be a serious public health issue in Rwanda, particularly for expecting mothers. The results of the study demonstrate the critical need for focused treatments to address this problem and enhance the dental health of Rwandan pregnant women [24]. Likewise, another study conducted on Indonesian women reported the prevalence of periodontitis to be 48.5% [3]. A study conducted in India reported the prevalence of periodontitis in pregnant women to be 56.8% [25]. Similarly, a study conducted in Pakistan reported that 34.5% of pregnant women were diagnosed with periodontitis. Although this prevalence rate is lower compared with the other countries mentioned, it still represents a significant portion of the pregnant population affected by this condition. The findings from Pakistan call for increased awareness and preventive measures to mitigate the impact of periodontitis on maternal and fetal health [26].

These studies demonstrate the worldwide character of periodontitis in pregnant women, emphasizing the necessity for universal measures to tackle the problem. Prevalence rates vary among nations due to differences in healthcare infrastructure, availability to dental treatment, socioeconomic considerations, and cultural attitudes toward oral health [27, 28]. Dental practitioners play an important role in providing preventative care and treatment that is targeted to the specific requirements of pregnant women.

### 2.3. Pregnancy Tumors (Pyogenic Granuloma)

Pregnancy tumors are benign growths that can occur in the oral cavity during pregnancy. They are often referred to as pyogenic granulomas or granuloma gravidarum in clinical settings. Usually, they appear as little, crimson nodules on the gums that are prone to bleeding and frequently sensitive to touch. These tumors affect about 5% of pregnant women, and they are more common during pregnancy [29]. Although the precise reason is unknown, it is thought to be connected to hormonal changes, including elevated levels of progesterone and estrogen, which can exacerbate the way oral tissues react to nearby irritants such as calculus and plaque. Maintaining good oral hygiene and receiving routine dental treatment are essential to controlling and avoiding pregnancy-related issues [30]. A clinicopathological analysis conducted in India reported that nearly 90% of the cases of pregnancy tumors occur on the gums as pyogenic granulomas [31].

### 2.4. Tooth Erosion

Tooth erosion, which is the loss of tooth hard tissue due to acid without bacterial involvement, is a major concern during pregnancy. This disease is sometimes aggravated by increased exposure to stomach acids caused by morning sickness and gastroesophageal reflux, which are prevalent during pregnancy. Research shows that about 30%–50% of pregnant women report experiencing morning sickness, which contributes to enamel erosion [32]. Research indicates that pregnant women commonly experience increased rates of tooth erosion in comparison to nonpregnant women, with prevalence rates varying widely but consistently highlighting a notable impact on oral health during pregnancy. Hormonal changes during pregnancy can also alter saliva composition and flow, reducing its buffering capacity and increasing the risk of erosion [33, 34]. Effective management includes dietary counseling, encouraging oral hygiene practices, and using protective agents like fluoride to mitigate the effects of acid exposure. In addition, the altered dietary habits during pregnancy, which often involve a greater desire for acidic foods, can further increase the risk of tooth erosion [35].

### 2.5. Tooth Decay

Tooth decay, or dental caries, is a common issue during pregnancy due to a variety of physiological and behavioral changes. Pregnant women are more likely to develop tooth decay due to hormonal changes that might impact the oral environment, such as decreased saliva flow and changed saliva composition, which weaken the mouth's natural defense against germs. Morning sickness, characterized by frequent vomiting, releases stomach acids into the mouth cavity, eroding enamel and providing an ideal habitat for decay-causing bacteria. Furthermore, pregnant cravings for sweet meals and carbs increase the risk of caries [36]. A study conducted in Colombia reported that 89.9% of pregnant women had caries [37]. Another study conducted in Brazil reported that the prevalence of dental caries in pregnant women is 60% [4]. Routine dental checkups, good oral hygiene habits such as brushing and flossing with fluoride toothpaste, and dietary changes to minimize sugar intake are all effective ways to control tooth decay during pregnancy.

## 3. Adverse Pregnancy Outcomes Associated With Poor Oral Health

Poor oral health during pregnancy is linked to several unfavorable outcomes that can negatively impact both maternal and fetal health. One major concern is the increased risk of delivering low-birth-weight babies, defined as infants weighing less than 2500 g [38]. In addition, poor oral health can contribute to preterm birth, which occurs when a baby is born before 37 weeks of gestation. Preeclampsia, another serious condition associated with poor oral health, involves high blood pressure and potential organ damage, posing significant risks to both the mother and the fetus. Gestational diabetes is another complication that can arise, further complicating pregnancy and delivery. These adverse outcomes are often the result of severe dental infections, such as periodontitis. Periodontitis can release harmful substances such as prostaglandins and cytokines into the bloodstream, which can disrupt glucose metabolism, hinder fetal development, and trigger early labor [39–41]. The detail can be seen in Table 1.

### 3.1. Preterm Birth

Preterm birth risk has been strongly correlated with poor dental health, especially periodontal disease. Prostaglandins and cytokines are examples of inflammatory mediators that can be released systemically in response to periodontitis, a severe kind of gum disease that involves persistent inflammation and infection of the gums and surrounding tissues. These substances are known to contribute to the onset of labor, which may lead to an early delivery occurring before 37 weeks of pregnancy [47]. It has been noted that prostaglandin inhibitors are used to delay and prevent the onset of early labor. Elevated PGE2 levels are negatively correlated with clinical attachment levels and probing depth, two key periodontal indicators [48]. Also, research has repeatedly shown that the degree of periodontal deterioration in moms who also occurred to be more prone to give birth prematurely is positively correlated with PGE2 levels in obtained GCF samples [9]. In human case-control research, Offenbacher et al. were the first to record a connection between periodontal disorders and premature birth. When compared with women in a control group who carried out a full-term gestational period, they found that moms who delivered preterm low-birth-weight (PLBW) infants had considerably poorer periodontal disease conditions [49]. After this research, another study conducted by the same authors revealed a noteworthy rise in the incidence of preterm births (less than 28 weeks) in correlation with the mother's declining periodontal health [50]. A study conducted in India indicated that 15.87% of pregnant women diagnosed with periodontitis experienced preterm births [51]. Similarly, likewise, a separate study conducted in Ethiopia found that the prevalence of preterm births was 25.9% among pregnant women diagnosed with periodontitis [52]. Another study conducted in India found that at least 18% of pregnant women who experienced preterm birth had a history of periodontal disease [53]. Similarly, a study done in Italy also found a substantial link between periodontal disease and poor pregnancy outcomes in women over the age of 40. The findings highlight the need of routine oral health checkups and periodontal evaluations in older pregnant women to reduce the risks of preterm delivery and low birth weight. Integrating periodontal care into prenatal treatment may improve mother and newborn outcomes [54]. Therefore, maintaining good oral hygiene and managing periodontal health during pregnancy are crucial for reducing the risk of preterm birth and ensuring better pregnancy outcomes.

### 3.2. Low Birth Weight

PLBW continues to be a serious public health concern. Affected infants are more likely to experience epilepsy, cerebral palsy, respiratory distress syndrome, pathologic cardiac diseases, and significant learning difficulties [55]. Public health strategies have been used to target PLBW in a number of epidemiologic investigations [56]. Despite breakthroughs in pharmaceuticals that can stop premature labor and a greater understanding of reproductive physiology, the rate of preterm births in the West seems to be rising. Low birth weight has been associated with poor maternal dental health, including periodontal disease. Periodontal disease is associated with chronic inflammation, which may trigger inflammatory responses in the body, potentially affecting fetal development and birth outcomes [43]. The relationship between adverse pregnancy outcomes and periodontitis can be understood through two mechanisms. First, periodontal pathogens such as *Porphyromonas gingivalis*, *Fusobacterium nucleatum*, *Prevotella intermedia*, *Aggregatibacter actinomycetemcomitans* (A.A), and *Treponema denticola* can directly migrate to the fetoplacental unit (Table 2). Second, inflammatory mediators like interleukin-1 (IL-1), IL-6, IL-8, tumor necrosis factor-alpha (TNF-α), and prostaglandin E2 (PGE2) can indirectly provoke a systemic response. This response prompts the liver to increase the production of C-reactive protein and fibrinogen, both of which play a role in inflammation [64]. A study conducted in India found that 11.4% of the sample population had low birth weight due to periodontitis [65]. A systematic review conducted in Ethiopia in 2015 suggested a correlation between periodontal disease and a higher risk of preterm birth, low birth weight, or both [43]. According to research, pregnant women with untreated periodontal disease had a much higher risk of having low birth weight kids than those with healthy gums. Periodontal bacteria can harm fetal growth and development by infecting the placenta and causing an immune response. Maintaining good oral hygiene and obtaining regular dental treatment during pregnancy can assist to reduce these risks and promote healthier pregnancy outcomes [66]. Conversely, a Spanish study by Moreu et al. found a statistically significant positive correlation between PLBW and deeper maternal probing. Although they failed to find a link between maternal periodontal disease and preterm delivery, they nevertheless regarded it as a risk factor for PLBW [67] (Table 2).

### 3.3. Preeclampsia

Preeclampsia, a dangerous pregnancy complication defined by high blood pressure and evidence of damage to other organ systems, most commonly the liver and kidneys, has been associated to poor dental health. According to research, there is a substantial link between periodontal disease (a serious gum infection) and an increased chance of getting preeclampsia while pregnant [68]. Periodontal disease has systemic implications, including the generation of inflammatory cytokines and other indicators associated with preeclampsia. These inflammatory reactions can impair the body's capacity to control blood pressure and sustain good pregnancy circumstances [69]. A study conducted in Iran reported that 59.54% of pregnant women with preeclampsia showed signs of periodontitis [70]. Maintaining proper dental hygiene and resolving periodontal disorders early in pregnancy can significantly reduce the incidence of preeclampsia, resulting in better health outcomes for both mother and baby.

### 3.4. Gestational Diabetes

Gestational diabetes mellitus (GDM) is a disorder that causes high blood glucose levels during pregnancy, posing dangers to both the mother and the baby. Emerging research indicates a strong association between poor dental health and the occurrence of gestational diabetes. Periodontal disease, a chronic inflammatory illness affecting the gums and supporting tissues of the teeth, has been identified as a possible risk factor for GDM [71]. The systemic inflammation caused by periodontal disease can affect glucose metabolism, resulting in insulin resistance, which is a major risk factor for gestational diabetes. Furthermore, oral infections might aggravate inflammatory responses, raising the risk of GDM [72]. A study conducted in India reported that the prevalence of periodontitis was 40% among women with GDM, suggesting a potential association between periodontitis and GDM. This significant prevalence highlights the importance of considering oral health as a contributing factor in the development and management of gestational diabetes [25]. Another study conducted in Finland reported that 32% of pregnant women with GDM had severe periodontitis [72]. Given this high incidence, addressing oral health concerns during pregnancy is critical not just for avoiding dental disorders but also for lowering the risk of gestational diabetes and its complications. Regular dental checkups, keeping good oral hygiene, and effectively controlling periodontal disease can play an important role in enhancing overall maternal health and minimizing the risks of GDM.

### 3.5. Miscarriage

Poor dental health, particularly severe periodontal disease, is associated with an increased risk of miscarriage. Periodontal disease is a chronic inflammatory disorder that affects the gums and the bone structures that support the teeth. Periodontal disease can cause systemic inflammation and bacterial infection, leading to the generation of inflammatory mediators such as prostaglandins and cytokines. These inflammatory mediators have the potential to alter the uterine environment and cause pregnancy problems [73]. A study conducted in Thailand reported that periodontitis was present in 50.6% of women who experienced miscarriages, compared with 21.2% of women in the control group [74]. A recent study on American and Canadian women found that 10% had a history of periodontitis diagnosis at the time of miscarriage [75]. This significant difference suggests a potential association between periodontal disease and an increased risk of adverse pregnancy outcomes. However, further clinical trials and advanced studies are also required to support and validate the relationship between the effects of oral health on adverse pregnancy outcomes. Therefore, it is imperative to have routine dental examinations as part of comprehensive prenatal care that can help detect and treat periodontal disease early on, which lowers the risk of miscarriage and other unfavorable pregnancy outcomes.

In conclusion, preserving good oral health throughout pregnancy is critical to avoiding unfavorable pregnancy outcomes. Periodontal disease, gingivitis, and other oral infections are the significant but modifiable risk factors for unfavorable pregnancy outcomes, as they can greatly raise the risk of complications such as low birth weight, preterm birth, preeclampsia, gestational diabetes, and miscarriage. These conditions set off systemic inflammation and immune responses that can impede fetal development and maternal health. Consequently, it is critical to minimize these risks and promote healthier pregnancy outcomes by ensuring pregnant women maintain good oral hygiene and receive regular dental care. Comprehensive prenatal care should include routine dental evaluations and treatments to protect the health of both mother and fetus.

## Figures and Tables

**Table 1:** Key adverse pregnancy outcomes associated with poor oral health.

Adverse pregnancy outcome	Description	Associated oral health condition	Mechanism	References
Preterm birth	Delivery before 37 weeks of gestation	Periodontitis, gingivitis	Inflammation triggers release of prostaglandins and cytokines, inducing labor	[42]
Low birth weight	Birth weight less than 2500 g (5.5 pounds)	Periodontitis	Systemic inflammation affects fetal growth	[43]
Preeclampsia	High blood pressure and organ damage, often kidneys	Periodontitis	Systemic inflammatory response contributes to endothelial dysfunction	[44]
Gestational diabetes	High blood sugar during pregnancy	Periodontitis	Inflammation affects glucose metabolism	[45]
Miscarriage	Loss of pregnancy before 20 weeks	Severe oral infections	Systemic inflammation and infection spread	[46]

**Table 2:** Periodontal pathogens linked to adverse pregnancy outcomes.

Pathogens	Findings	References
*P. micros*, *C. rectus*	*P. micros* and *C. rectus* were associated with PLBW	[57]
*F. nucleatum*	Omega-3 fatty acids suppress inflammatory responses elicited by bacteria, making them potentially effective in reducing adverse pregnancy outcomes	[58]
*F. nucleatum*	*F. nucleatum* can translocate from the oral to the uterine environment	[59]
*F. nucleatum*	The hematologic transmission capabilities of *F. nucleatum* could induce adverse pregnancy outcomes	[60]
*P. gingivalis*	The pathogenesis of *P. gingivalis* is associated with adverse pregnancy outcomes: maternal/fetal tissue alterations via the action of virulence factors, surface adhesion molecules, and enzymes; enhanced production of cytokines, oxidative stresses, and acute-phase proteins; and elevated fetal adrenal cortisol levels	[61]
*P. gingivalis*	The pathogenesis of *P. gingivalis* is associated with adverse pregnancy outcomes: maternal/fetal tissue alterations via the action of virulence factors, surface adhesion molecules, and enzymes; enhanced production of cytokines, oxidative stresses, and acute-phase proteins; and elevated fetal adrenal cortisol levels	[62]
*P. gingivalis*, *Tannerella forsythia*, *Prevotella intermedia*, *Prevotella nigrescens*, A.A	Statistically significant higher levels of all bacteria associated with preterm births in comparison with full-term births; A 2.4-fold increase in levels of A.A bacteria in preterm deliveries as the pregnancy progresses until after birth when compared with full-term deliveries	[63]

## Data Availability

The data used to support the findings of this study are available on request from the corresponding authors.
