# Correction to “Exploring the Influence of Oral Health on Pregnancy Outcomes: A Narrative Review”

**DOI:** 10.1155/ghe3/9757858

**Published:** 2026-09-22

M. M. Javaid, S. N. Khalid, S. A. Khan, et al., “Exploring the Influence of Oral Health on Pregnancy Outcomes: A Narrative Review,” *Global Health, Epidemiology and Genomics* 2025 (2025): 9304496, https://doi.org/10.1155/ghe3/9304496.

In the article titled “Exploring the Influence of Oral Health on Pregnancy Outcomes: A Narrative Review,” an affiliation for Muhammad Mohsin Javaid was inadvertently omitted. The missing affiliation has now been added as Affiliation 2. In addition, “Department” has been changed to “School” in affiliation 1. Accordingly, the authors’ affiliation information has been updated as follows:

Muhammad Mohsin Javaid^1,2^ | Samina Naeem Khalid^3^ | Shahzad Ali Khan^1^ | Hina Nasim^4^ | Mudassar Mushtaq Jawad Abbasi^1^ | Shahzad Ahmad^5^ | Shazia Iqbal^5^ | and Muhammad Farooq Umer^6^

^1^School of Public Health, Health Services Academy, Islamabad, Pakistan

^2^Department of Community and Preventive Dentistry, School of Dentistry, Shaheed Zulfiqar Ali Bhutto Medical University Islamabad, Pakistan

^3^Department of Maternal, Neonatal & Child Health, Health Services Academy, Islamabad, Pakistan

^4^Department of Oral Biology, School of Dentistry/SZABMU, Islamabad, Pakistan

^5^Faculty of Medicine and Health Sciences, The University of Buckingham, Buckingham, UK

^6^Department of Preventive Dental Sciences, College of Dentistry, King Faisal University, AlAhsa, Hofuf 31982, Saudi Arabia

We apologize for this error.

